# The Roth score as a triage tool for detecting hypoxaemia in general practice: a diagnostic validation study in patients with possible COVID-19

**DOI:** 10.1017/S1463423621000347

**Published:** 2021-10-18

**Authors:** Charlotte E.M. ten Broeke, Jelle C.L. Himmelreich, Jochen W.L. Cals, Wim A.M. Lucassen, Ralf E. Harskamp

**Affiliations:** 1 Department of General Practice, Amsterdam UMC, University of Amsterdam, Amsterdam Cardiovascular Sciences and Amsterdam Public Health, Amsterdam, the Netherlands; 2 Department of Family Medicine, Maastricht University, Maastricht, the Netherlands

**Keywords:** COVID-19, general practice, hypoxaemia, pulse oximetry, Roth score, SARS-CoV-2

## Abstract

**Aim:**

To validate the Roth score as a triage tool for detecting hypoxaemia.

**Backgrounds:**

The virtual assessment of patients has become increasingly important during the corona virus disease (COVID-19) pandemic, but has limitations as to the evaluation of deteriorating respiratory function. This study presents data on the validity of the Roth score as a triage tool for detecting hypoxaemia remotely in potential COVID-19 patients in general practice.

**Methods:**

This cross-sectional validation study was conducted in Dutch general practice. Patients aged ≥18 with suspected or confirmed COVID-19 were asked to rapidly count from 1 to 30 in a single breath. The Roth score involves the highest number counted during exhalation (counting number) and the time taken to reach the maximal count (counting time).

Outcome measures were (1) the correlation between both Roth score measurements and simultaneous pulse oximetry (SpO_2_) on room air and (2) discrimination (c-statistic), sensitivity, specificity and predictive values of the Roth score for detecting hypoxaemia (SpO_2_ < 95%).

**Findings:**

A total of 33 physicians enrolled 105 patients (52.4% female, mean age of 52.6 ± 20.4 years). A positive correlation was found between counting number and SpO_2_ (*r*_s_ = 0.44, *P* < 0.001), whereas only a weak correlation was found between counting time and SpO_2_ (*r*_s_ = 0.15, *P* = 0.14). Discrimination for hypoxaemia was higher for counting number [c-statistic 0.91 (95% CI: 0.85–0.96)] than for counting time [c-statistic 0.77 (95% CI: 0.62–0.93)]. Optimal diagnostic performance was found at a counting number of 20, with a sensitivity of 93.3% (95% CI: 68.1–99.8) and a specificity of 77.8% (95% CI: 67.8–85.9). A counting time of 7 s showed the best sensitivity of 85.7% (95% CI: 57.2–98.2) and specificity of 81.1% (95% CI: 71.5–88.6).

**Conclusions:**

A Roth score, with an optimal counting number cut-off value of 20, maybe of added value for signalling hypoxaemia in general practice. Further external validation is warranted before recommending integration in telephone triage.

## Introduction

In the current coronavirus disease (COVID-19) pandemic, the first assessment of patients presenting with respiratory symptoms is virtually always performed by telephone (Greenhalgh *et al.*, 2020a, Greenhalgh *et al.*, 2020b, Smith *et al.*, 2020). Furthermore, monitoring of COVID-19 is also primarily done remotely, unless progression of symptoms warrants in-person evaluation (Greenhalgh *et al.*, 2020b, Greenhalgh *et al.*, 2020a, Smith *et al.*, 2020, Huang *et al.*, 2020). In order to assess whether COVID-19 deteriorates, telephone assessment focusses on deteriorating respiratory function (Greenhalgh *et al.*, 2020a, The Centre for Evidence-Based Medicine, 2020b, Huang *et al.*, 2020, The Centre for Evidence-Based Medicine, 2020a). Unfortunately, an accurate assessment of the patient’s respiratory status can be challenging during telephone triage, as patients with COVID-19 may be hypoxemic without presenting with typical warning signs, such as dyspnoea (O’Driscoll *et al.*, 2017, Huang *et al.*, 2020, Greenhalgh *et al.*, 2020a, The Centre for Evidence-Based Medicine, 2020a, The Centre for Evidence-Based Medicine, 2020b, Ottestad *et al.*, 2020, Teo, 2020, Chen *et al.*, 2020, Xie *et al.*, 2020). Early on in the pandemic, a simple breathing test was therefore promoted through medically oriented social media channels to assist in the detection of hypoxaemia during telephone triage (Chorin *et al.*, 2016, The Centre for Evidence-Based Medicine, 2020b). This breathing test, named as the ‘Roth score’, was developed in an in-hospital population of patients with cardiopulmonary pathology and was based on a patient’s ability to count up to 30 in a single exhalation (counting number), as well as the time taken to reach a maximal count in seconds (counting time) (Chorin *et al.*, 2016). This score showed a strong positive correlation with pulse oximetry and good discrimination for detecting hypoxaemia. However, while promising, the Roth score was never externally validated, and certainly not in a community-based setting (Chorin *et al.*, 2016, The Centre for Evidence-Based Medicine, 2020b, The Centre for Evidence-Based Medicine, 2020a). Providing primary care physicians and telephone triagists with a reliable score to be used in the remote assessment of possible hypoxemic patients could be crucial in the ongoing COVID-19 pandemic. The purpose of the current study was, therefore, to determine the diagnostic accuracy of the Roth score as a tool assisting in the diagnosis of hypoxaemia, compared with pulse oximetry as the reference standard, in suspected COVID-19 patients in general practice.

## Methods

We reported the methods and findings of this study confirm to the Standards for Reporting of Diagnostic Accuracy Studies (STARD 2015) (Bossuyt *et al.*, 2015).

### Study design

We performed a cross-sectional, clinical validation study in which we asked general practitioners (GPs), situated in primary care practices throughout the Netherlands, to evaluate patients for eligibility and enrol patients during consultation. GPs were approached to participate both structurally, by coordinating healthcare organisations, and personally, through word-of-mouth referral and social network channels. As such, recruitment of physicians occurred via virtual snowball sampling. For each patient, the index test, reference standard and data collection were performed in a single consultation. Given the cross-sectional nature of this study, no follow-up data were collected.

### Study sample

Eligible patients were at least 18 years of age who were presented with symptoms suggestive for or caused by a confirmed severe acute respiratory syndrome coronavirus (SARS-CoV-2) infection, and was able to perform the Roth test.

We estimated the prevalence of patients with simultaneous pulse oximetry (SpO_2_) < 95% to be approximately 15% in general practice, which requires a minimal sample of 155 subjects to achieve a minimum power of 80% to detect a change in the percentage value of sensitivity from 0.70 to 0.90, based on a target significance level of 0.05. This sample size is also sufficient to detect a change specificity value from 0.70 to 0.90, which requires a sample of 39 subjects (Bujang and Adnan, 2016).

### The Roth score (index test)

Patients were instructed to take a deep breath and subsequently count out loud, as fast as possible, from 1 to 30, during a single exhalation. The Roth score includes two measurements: (a) the counting time as a measure for the duration of time in seconds between counting from 1 to 30 in one breath, or until the next inhalation and (b) the counting number as a measure for the highest number counted in one breath (Chorin *et al.*, 2016). In the derivation study, a maximal counting number <15 and a counting time <8 s were found to be optimal for identifying patients with a room air SpO_2_ < 95% (Chorin *et al.*, 2016).

### Pulse oximetry (reference standard) and definition of hypoxaemia

Peripheral oxygen saturation (SpO_2_) on room air, measured by validated pulse oximeters, was used as the reference standard for the detection of hypoxaemia in this study. This measurement is non-invasive, easily executable in general practice and provides a reflection of the true arterial oxygen saturation (SaO_2_) as determined by arterial blood gas (ABG) analysis, which is the golden standard for detecting hypoxaemia (O’Driscoll *et al.*, 2017).

Since there is no exact threshold of oxygen saturation below which a patient becomes hypoxemic, the threshold varies between a SpO_2_ of 90% and 95% amongst studies (Kelly *et al.*, 2001, O’Driscoll *et al.*, 2017, Greenhalgh *et al.*, 2020a). We focused on a SpO_2_ of 95% as the primary cut-off value, as saturation levels below this threshold are considered high risk for respiratory deterioration in patients without pre-existing pulmonary disease. Second, we also determined the diagnostic accuracy for identifying SpO_2_ < 90%, in accordance with the derivation study (Chorin *et al.*, 2016).

### Data collection

GPs entered data by using an electronic case report form (eCRF) that was accessible through a dedicated website (https://www.rothscore.nl). Instructions regarding the execution of the test were provided both textually and by video demonstration on the website. The eCRF consisted of index and reference standard measurements, demographic data, clinical manifestations, vital parameters, underlying comorbidities, the use of immunosuppressant therapy, smoking status and COVID-19 exposure history. The likelihood of SARS-CoV-2 infection was left at the discretion of the participating clinician, either based on a SARS-CoV-2 polymerase chain reaction (PCR) test or based on clinical suspicion.

### Outcomes of interest

We studied the correlation between the Roth score, consisting of counting time and counting number, and SpO_2_ on room air, with pulse oximetry as the reference standard. Subsequently, we assessed the diagnostic accuracy measures of discrimination (c-statistic), sensitivity, specificity, the positive and negative predictive values of the Roth score as an instrument for detecting hypoxaemia (SpO_2_ < 95% and <90%).

### Statistical analysis

The baseline characteristics are described as proportions, means or medians with corresponding dispersion measures. We displayed the correlations between counting number, counting time and SpO_2_ on room air in scatter plots. We computed correlation coefficients with corresponding 95% confidence intervals (CIs) by using bias-corrected and accelerated (BCa) bootstrapping. Correlations of 0.1 were considered weak, 0.4 moderate and 0.7 strong (Akoglu, 2018). We constructed a Receiver Operating Characteristic (ROC) curve and computed the c-statistic with corresponding 95% CI to present the discriminatory ability of the index test. The Roth score’s diagnostic accuracy for detecting SpO_2_ < 95% and <90% was determined by calculating the sensitivity (SENS), specificity (SPEC), positive predictive value (PPV) and negative predictive value (NPV) for the cut-off values of counting number and counting time with 95% CIs. Statistical analyses were performed by using IBM SPSS 26.0 and MedCalc 2020.

## Results

### Patient flow and baseline characteristics

**Figure 1:**
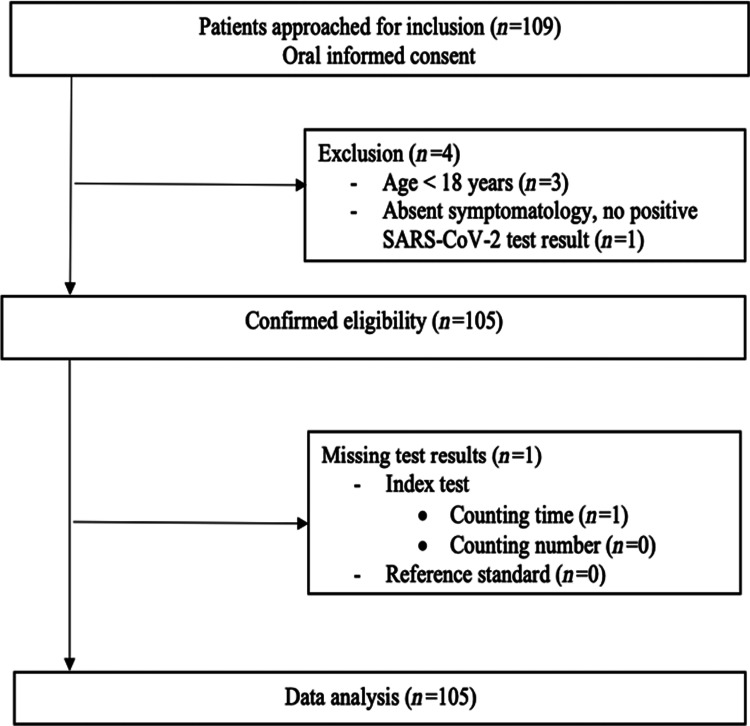
Flowchart of patient selection.

A total of 109 patients were enrolled in this study by 33 independent GPs between 4 April 2020 and 19 June 2020. Of those, four patients did not meet the eligibility criteria and were excluded from data analysis (Figure 1). The final study population consisted of 105 individuals (52.4% female, mean age of 52.6 ± 20.4 years), from whom the baseline characteristics are shown in Table 1. The majority of patients (*n* = 91, 90.1%) presented in GP offices, while 10 patients (9.9%) presented in out-of-hours GP urgent care centres. Of all patients, 11 (10.5%) were known PCR positive for SARS-CoV-2 at the time of presentation, whereas the diagnosis was considered likely by the assessing physician in 53 (50.5%) patients. The predominant presenting symptoms were coughing (61%), dyspnoea (58.1%) and exhaustion (56.2%). The most frequent occurring comorbidities were hypertension (31.4%), pulmonary disease (23.8%) and diabetes mellitus (10.5%). The median oxygen saturation at presentation was 98.0% [interquartile range (IQR) 96.5–98.5], heart rate 84.0 bpm (IQR 72.8–97.0), respiratory rate 16.0 breaths/min (IQR 14.0–18.0), body temperature 37.1°C (IQR 36.7–37.7). Of all included patients, a total of 15 individuals (14.3%) had a SpO_2_ < 95%, of which 4 individuals (3.8%) <90% (Table 2). *Supplementary Table 1* shows the distribution of patients over the counting number and counting time categories.

**Table 1.** Characteristics of study participants (*n* = 105)

Variable		Mean ± SD, median (25th–75th) or number (%)
Age (years)		52.6 ±20.4
Gender (female)		54/103 (52.4)
Symptoms	Cough	64 (61.0)
	Dyspnoea	61 (58.1)
	Exhaustion	59 (56.2)
	Fever	24 (22.9)
	Sore throat	16 (15.2)
	Chest pain	16 (15.2)
	Rhinitis	15 (14.3)
	Myalgia	7 (6.7)
	Other	16 (15.2)
Vital parameters	Oxygen saturation (%)	98.0 (96.5–98.5)
	Heart rate (bpm)	84.0 (72.8–97.0)
	Respiratory rate (breaths/min)	16.0 (14.0–18.0)
	Body temperature (°C)	37.1 (36.7–37.7)
Comorbidities	Hypertension	33 (31.4)
	COPD, asthma	25 (23.8)
	Diabetes mellitus	11 (10.5)
	Coronary heart disease	9 (8.6)
	Heart failure	5 (4.8)
	Obesity	7 (6.7)
	Atrial fibrillation	3 (2.9)
	Lung carcinoma	3 (2.9)
	Kidney disease	3 (2.9)
	Sarcoidosis	2 (1.9)
	Pulmonary embolism	2 (1.9)
	Chronic rhinosinusitis	2 (1.9)
	Hypercholesterolemia	2 (1.9)
	Valvular disease	2 (1.9)
	Other	6 (5.7)
Therapy	Immunosuppressants	3 (2.9)
Smoking (current)		11 (10.5)
COVID-19 status	Confirmed	11 (10.5)
	Likely positive	53 (50.5)
	(Likely) post-COVID-19	2 (1.9)
	Unlikely	39 (37.1)
Setting of presentation	GP office/home visitation	91/101 (90.1)
	GP urgent care centre/home visitation	10/101 (9.9)

SD = standard deviation; 25th = first quartile; 75th = third quartile; bpm = beats per minute; min = minute; °C = degrees Celsius; COPD = Chronic Obstructive Pulmonary Disease; COVID-19 = coronavirus disease 2019; GP = General Practitioner.

**Table 2.** Counting number and counting time stratified by hypoxaemia status

	SpO_2_ ≥ 95%(*n* = 90)	SpO_2_ < 95%(*n* = 15*)
**Counting number** Median (25th–75th)	30.0 (21.0–30.0)	17.0 (14.0–19.0)
**Counting time** Mean** ±** SD	9.3 ± 2.8 s	7.2 ± 2.7 s

SpO_2_ = peripheral oxygen saturation; *N* = number; *, of which 4 < 90%; 25th = first quartile; 75th = third quartile; SD = standard deviation.

### Correlation between the Roth score and pulse oximetry

**Figure 2.**
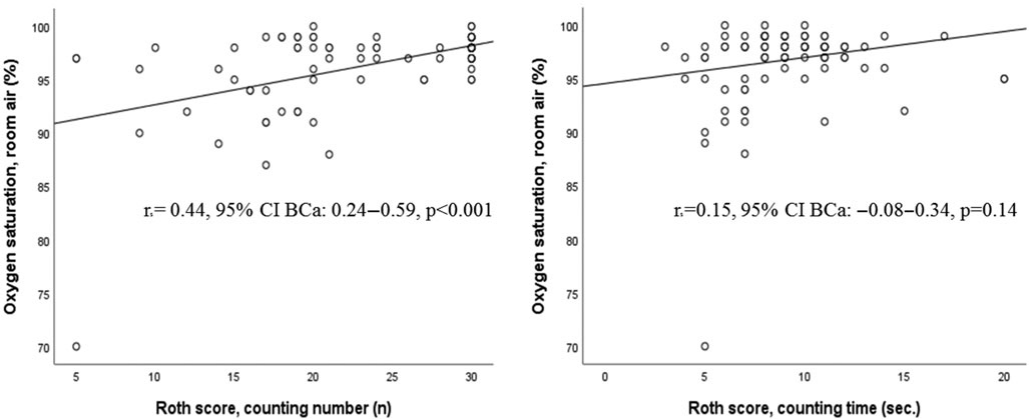
Correlation plots of the Roth score (counting number: left panel; counting time: right panel) and SpO_2_ on room air.

Figure 2 shows the correlations between counting number, counting time and SpO_2_ on room air. The correlation analysis showed a moderately positive, linear correlation between counting number and pulse oximetry, and a weak positive, linear correlation between counting time and pulse oximetry.

### Diagnostic accuracy of the Roth score

**Figure 3.**
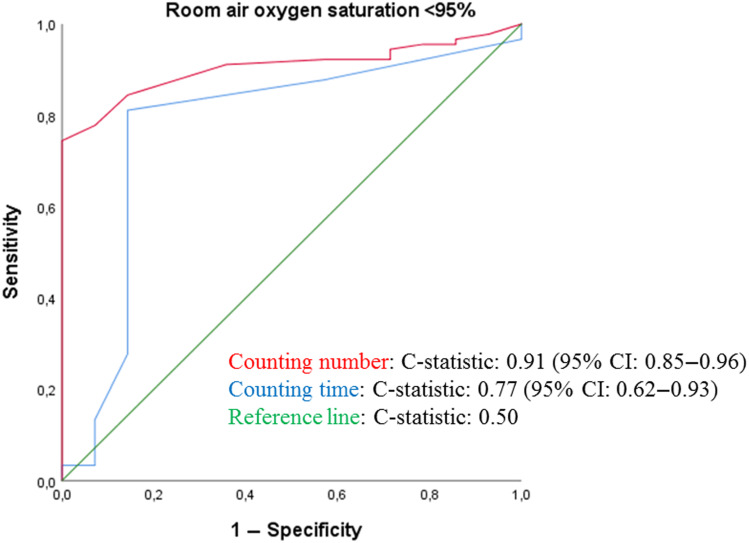
ROC curve assessing the discriminatory ability of the Roth score for identifying SpO_2_ < 95%.

As shown in Table 2, patients with a SpO_2_ ≥ 95% had higher median counting number and mean counting time, compared to those with lower SpO_2_ values. Figure 3 shows the discrimination plots of hypoxaemia for counting number (c-statistic: 0.91) and counting time (c-statistic: 0.77), respectively. Diagnostic accuracy in terms of SENS and SPEC are shown in Table 3 and *Supplementary* Table 2. Of all tested cut-off values, optimal accuracy was found for a counting number of 20 (SENS 93.3%, SPEC 77.8%, PPV 41.2%, NPV 98.6%) and a counting time of 7 s (SENS 85.7%, SPEC 81.1%, PPV 41.4%, NPV 97.3%).

**Table 3.** Diagnostic accuracy of the Roth score

	Oxygen saturation <95%				Oxygen saturation <90%			
	SENS (%)	SPEC (%)	PPV (%)	NPV (%)	SENS (%)	SPEC (%)	PPV (%)	NPV (%)
Max. CN								
7	6.7	97.8	33.3	86.3	25.0	98.0	33.3	97.1
10	13.3	95.6	33.3	86.9	25.0	95.1	16.7	97.0
15	26.7	92.2	36.4	88.3	50.0	91.2	18.2	97.9
16	40.0	92.2	46.2	90.2	50.0	89.1	15.4	97.8
17	66.7	91.1	55.6	94.3	75.0	85.1	16.7	98.9
18	73.3	88.9	52.4	95.3	75.0	82.2	14.3	98.9
19	86.7	84.4	48.2	97.4	75.0	76.2	11.1	98.7
20	93.3	77.8	41.2	98.6	75.0	69.3	8.8	98.6
Max. CT (s)								
5	21.4	92.2	30.0	88.3	66.7	92.1	20.0	98.9
6	42.9	87.8	35.3	91.0	66.7	85.1	11.8	98.9
7	85.7	81.1	41.4	97.3	100	74.3	10.3	100
8	85.7	62.2	26.1	96.6	100	57.4	6.5	100
9	85.7	44.4	19.4	95.2	100	41.6	4.8	100
10	85.7	27.8	15.6	92.6	100	26.7	3.9	100
11	92.9	13.3	14.3	92.3	100	12.9	3.3	100
12	92.9	7.8	13.5	87.5	100	7.9	3.1	100
13	92.9	5.6	13.3	83.3	100	5.9	3.1	100

SENS = sensitivity; SPEC = specificity; PPV = positive predictive value; NPV = negative predictive value; max = maximum; s = seconds; CN = counting number; CT = counting time.

## Discussion

### Key findings

In this study, we assessed the diagnostic accuracy of the Roth score as an instrument for assisting in the detection of hypoxaemia in patients with SARS-CoV-2 symptoms in a primary care-based setting during the early phase of the COVID-19 pandemic. Our study showed good discriminatory ability of counting number and counting time for identifying hypoxaemia (SpO_2_ < 95%). The optimal cut-off value for identifying a room air oxygen saturation <95% in terms of sensitivity, specificity and predictive values was found for a maximal counting number of 20 and a counting time of 7 s.

### Strengths and limitations

Our study provides important data on the validity of the Roth score in identifying patients with hypoxaemia in general practice. This is a relevant subject in the COVID-19 pandemic in which patients are more frequently assessed remotely. The use of social media channels for physician recruitment largely contributed to the expanse of the sample size and geographical scope of this study (Baltar and Brunet, 2012). Another strength lies in the simplicity of the number of items we asked physicians to record, resulting in nearly all patients receiving both the index test and the reference standard. Each patient received the same reference standard, directly after the index test, herby avoiding partial verification bias and condition progression bias.

Several limitations should be mentioned. During the inclusion phase of our study, the number of new COVID-19 cases decreased substantially (at the end of the first wave), which meant we could enrol 105 of the projected 155 patients. Consequentially, we were able to include a smaller number of patients with hypoxaemia, resulting in reduced precision of the diagnostic accuracy estimates of the Roth score.

Second, the use of snowball sampling for the recruitment of physicians may have introduced selection bias.

Third, we included a relatively young patient sample compared to age categories in which the highest COVID-19 morbidity and mortality are found potentially affecting our diagnostic accuracy results.

Fourth, we used pulse oximetry as the reference standard for hypoxaemia in this study. Although pulse oximeters reflect the SaO2 accurately at saturations above 88%, they are less reliable at lower oxygen saturation, potentially introducing misclassification bias (O’Driscoll *et al.*, 2017). Fifth, for the execution of both the index test and the reference standard, standardised instructions were provided on the website. However, given a large number of test performers, it is possible that small differences exist in the execution of both tests, possibly affecting estimates of diagnostic accuracy. Finally, the possible added value of the Roth score lies in telephone triage situations where pulse oximeter readings are unavailable. Our study, however, did not test the Roth score in a telephone triage setting, but instead during physical assessment.

### Prior studies

To our knowledge, the derivation study of the Roth score, led by Chorin et al, is the only prior study assessing the validity of the Roth score as a tool for detecting hypoxaemia (Chorin *et al.*, 2016). The participants of that study were markedly different from ours, as these involved patients admitted to a coronary care unit due to predominantly cardiac morbidity. Inherent to the clinical setting, they were able to assess a larger sample of patients with hypoxaemia; 65 patients and 22 patients with oxygen saturations <95% and <90%, respectively. Compared to our study, their findings showed stronger positive correlations between counting number and SpO_2_ (*r* = 0.67; *P* < 0.001), as well as between counting time and SpO_2_ (*r* = 0.59; *P* < 0.001). With a counting number’s AUC of 0.83 and a counting time’s AUC of 0.76 for identifying SpO_2_ < 95%, their findings showed comparable overall test performance of the Roth score (Chorin *et al.*, 2016). However, further evaluation of the diagnostic accuracy results showed a completely opposite gradient of sensitivity and specificity for the same cut-off values of counting number and counting time, resulting in different optimal cut-off values for daily clinical practice. They found a maximal counting number <15 and counting time <8 s to be associated with sensitivities of 83% and 78% and specificities of 71% and 71% for identifying hypoxaemia, respectively, (Chorin *et al.*, 2016). Unfortunately, we were not able to deduce the cause of these significant differences from their study design.

Resulting from the growing need for a tool that signals hypoxaemia remotely during the pandemic, the Roth score was advertised as an easily accessible telephone triage tool on multiple internet websites and was quickly incorporated into clinical guidelines (The Centre for Evidence-Based Medicine, 2020b). However, after GPs applied it inappropriately with negative consequences, the use of the score was dissuaded by the Oxford COVID-19 Evidence Service Team and the Royal College of General Practitioners (The Centre for Evidence-Based Medicine, 2020b, The Royal College of General Practitioners, 2020). They recommended an overall clinical assessment instead of using the Roth score for the remote assessment of hypoxaemia, due to its high false-positive and false-negative rate (The Centre for Evidence-Based Medicine, 2020a, The Centre for Evidence-Based Medicine, 2020b). Even though we believe that using solely the optimal cut-off value might diminish these errors and subsequent unwarranted policymaking, we would like to emphasise the importance of using the Roth score as part of an overall clinical assessment. The Roth score is not meant to substitute a holistic clinical assessment, but rather provides an additional tool that can contribute to the decision to direct a patient towards hospital admission.

### Implications for clinical practice and future directions

Current Dutch remote triage protocols in general practice focus on (alarm) symptoms, signs of haemodynamic instability and risk factors for complicated disease (KS, 2020). We regard the Roth score as an easily executable, low-resource and inexpensive instrument, which is potentially applicable to current telemedicine practice. Based on our results, a counting number with a cut-off value of 20 might be of additional value to signalling hypoxaemia remotely, although it likely will not provide sufficient accuracy to use as a replacement of an overall clinical assessment. Considering its sensitivity, we would advise in-person assessment for a counting number below 21 to rule out hypoxaemia, and provided that further clinical assessment does not give rise to in-person evaluation, conservative management if a patient counts higher than 20 in one breath.

We believe caution is warranted with using counting time in clinical practice since we found no significant correlation with pulse oximetry, and this measurement is subject to more external variables than counting number, subsequently affecting the clinical relevance of its result. Moreover, the measurement of solely counting number is more user-friendly and time-saving in clinical practice, where consultation time is limited and irrelevant proceedings are undesirable.

Finally, we believe that our study provides the groundwork for future studies to validate the Roth score in general practice. These studies should concentrate on external validation with larger sample sizes, older age categories and changes in the Roth score due to relevant cardiopulmonary comorbidities. Moreover, future studies should investigate the feasibility of the Roth score in a telemedicine setting, inter and intra user reproducibility and user-friendliness of the test. These studies are required before recommending the integration of the Roth score (i.e., counting number measurement) in triage protocols.

## Conclusion

The Roth score’s counting number, with an in-person assessment cut-off value of 20, is potentially of added value for signalling hypoxaemia remotely in patients with possible COVID-19 in general practice. We consider this measurement easily executable in general practice and potentially applicable to telemedicine practice. However, before recommending integration in the current triage protocols, external validation studies with larger sample sizes are warranted. We advise caution with using counting time in clinical practice, as we found no additional value of this measurement.

## Data Availability

All data relevant to the study are included in the article or uploaded as an online supplement. The datasets used during the current study are available from the corresponding author on reasonable request.

## Supplementary material

For supplementary material accompanying this paper visit http://dx.doi.org/10.1017/S1463423621000347. click here to view supplementary material
