# Supplementary material for: The Roth score as a triage tool for detecting hypoxaemia in general practice: a diagnostic validation study in patients with possible COVID-19
Source: Prim Health Care Res Dev. 2021 Oct 18;22:e56. doi: 10.1017/S1463423621000347 (PMC8527530; doi:10.1017/S1463423621000347)
Supplement: Supplementary file 1 [file S1463423621000347sup001.docx]

| Supplemental table 1. Number of participants per CN and CT category.  \|  \| Max. \| SpO2 ≥95%  n (%) \| SpO2 <95%  n (%) \| \| --- \| --- \| --- \| --- \| \| CN  Total \| 5-9 \| 3 (3.3) \| 2 (13.3) * \| \| 10-14 \| 2 (2.2) \| 2 (13.3) * \| \| 15-19 \| 9 (10.0) \| 9 (60.0) * \| \| 20-29 \| 21 (23.3) \| 2 (13.3) * \| \| 30+ \| 55 (61.1) \| 0 \| \|  \| 90 \| 15 \| \| CT (sec.)  Total \| 3-5 \| 7 (7.8) \| 3 (21.4) # \| \| 6-7 \| 10 (11.1) \| 9 (64.3) * \| \| 8-9 \| 33 (36.7) \| 0 \| \| 10-11 \| 28 (31.1) \| 1 (7.2) \| \| 12-20 \| 12 (13.3) \| 1 (7.2) \| \|  \| 90 \| 14 \|  Abbreviations: CN, counting number; CT, counting time; Max, maximum; N, number of patients; Sec, seconds; *, of which one <90%; # of which two <90%.Supplemental table 2. Sensitivity and specificity (incl. 95% CI) of the Roth score | | | | | | | | |
| --- | --- | --- | --- | --- | --- | --- | --- | --- | --- | --- | --- | --- | --- | --- | --- | --- | --- | --- | --- | --- | --- | --- | --- | --- | --- | --- | --- | --- | --- | --- | --- | --- | --- | --- | --- | --- | --- | --- | --- | --- | --- | --- | --- | --- | --- | --- | --- | --- | --- | --- |
|  | **Oxygen saturation <95%** | | | | **Oxygen saturation <90%** | | | |
|  | SENS (%) | 95% CI | SPEC (%) | 95% CI | SENS (%) | 95% CI | SPEC (%) | 95% CI |
| Max. CN |  | | | |  | | | |
| 7 | 6.7 | 0.2 – 32.0 | 97.8 | 92.2 – 99.7 | 25.0 | 0.6 – 80.6 | 98.0 | 93.0 – 99.8 |
| 10 | 13.3 | 1.7 – 40.5 | 95.6 | 89.0 – 98.8 | 25.0 | 0.6 – 80.6 | 95.0 | 88.8 – 98.4 |
| 15 | 26.7 | 7.8 – 55.1 | 92.2 | 84.6 – 96.8 | 50.0 | 6.8 – 93.2 | 91.1 | 83.9 – 95.9 |
| 20 | 93.3 | 68.1 – 99.8 | 77.8 | 67.8 – 85.9 | 75.0 | 19.4 – 99.4 | 69.3 | 59.3 – 78.1 |
| Max. CT (sec.) | | | | |  | | | |
| 5 | 21.4 | 4.7 – 50.8 | 92.2 | 84.6 – 96.8 | 66.7 | 9.4 – 99.2 | 92.1 | 85.0 – 96.5 |
| 6 | 42.9 | 17.7 – 71.1 | 87.8 | 79.2 – 93.7 | 66.7 | 9.4 – 99.2 | 85.1 | 76.7 – 91.4 |
| 7 | 85.7 | 57.2 – 98.2 | 81.1 | 71.5 – 88.6 | 100 | 29.2 - 100 | 74.3 | 64.6 – 82.4 |
| 8 | 85.7 | 57.2 – 98.2 | 62.2 | 51.4 – 72.2 | 100 | 29.2 - 100 | 57.4 | 47.2 – 67.2 |
| 9 | 85.7 | 57.2 – 98.2 | 44.4 | 34.0 – 55.3 | 100 | 29.2 - 100 | 41.6 | 31.9 – 51.8 |
| 10 | 85.7 | 57.2 – 98.2 | 27.8 | 18.9 – 38.2 | 100 | 29.2 - 100 | 26.7 | 18.4 – 36.5 |
| 11 | 92.9 | 66.1 – 99.8 | 13.3 | 7.1 – 22.1 | 100 | 29.2 - 100 | 12.9 | 7.0 – 21.0 |
| 12 | 92.9 | 66.1 – 99.8 | 7.8 | 3.2 – 15.4 | 100 | 29.2 - 100 | 7.9 | 3.5 – 15.0 |
| 13 | 92.9 | 66.1 – 99.8 | 5.6 | 1.8 – 12.5 | 100 | 29.2 - 100 | 5.9 | 2.2 – 12.5 |

Abbreviations: SENS, sensitivity; SPEC, specificity; CI, confidence interval; max, maximum; CN, counting number; CT, counting time; sec, seconds.
